# Sexual activities, erectile dysfunction, and the use of sexual management strategies among assigned males with genital ablation interests

**DOI:** 10.1093/sexmed/qfaf085

**Published:** 2025-10-28

**Authors:** Erik Wibowo, Thomas W Johnson, Richard J Wassersug, Jamie D Agapoff

**Affiliations:** School of Medical Sciences, Faculty of Medicine and Health, University of Sydney, The University of Sydney, NSW 2006, Australia; Department of Anthropology (Emeritus), California State University—Chico, CA 95929, United States; Department of Cellular and Physiological Sciences, University of British Columbia, Vancouver, BC V6T 1Z3, Canada; Department of Psychiatry, John A. Burns School of Medicine, University of Hawai’i at Manoa, Honolulu, HI 96813, United States

**Keywords:** Erectile dysfunction, Castration, Eunuchs, Sexual dysfunction, Sexuality, Sexual management strategies, Sex toys

## Abstract

**Background:**

Assigned males with genital ablation interests may be at higher risk of having erectile dysfunction (ED), especially those who have gone through androgen suppressing pharmacological therapies, orchiectomy and/or penectomy.

**Aim:**

To determine the prevalence and severity of ED in assigned males with castration interests, the types of management strategies they use and what factors are associated with using these strategies.

**Methods:**

We launched an online survey on the Eunuch Archive website to better understand how common ED is, as well as sexual frequencies, and the use of sexual management strategies among assigned males with genital ablation interests.

**Outcomes:**

ED and sexual activity frequencies, preferred role in sexual activities, and previous use of sexual management strategies.

**Results:**

Data from 363 assigned male individuals (50.7 ± 15.6 years old; 23.6% and 3.7% had been orchiectomized and penectomized respectively) showed that 11.2% reported having ED 25-50% of the time, 12.8% had ED 50-75% of the time, and 22.9% had ED 75-100% of the time. Yet a large proportion remained sexually active. For example, 63.5%, 56.2% and 8.8% reported watching porn, masturbating, and having partnered sex several times a week respectively. During partnered sex, 13% of the participants preferred to be in the insertive role, whereas nearly 40% preferred to be in the receptive role. A quarter preferred non-penetrative sex. To maintain sexual activities, commonly used strategies included oral medication (38.8%), vacuum erection devices (25.6%), and strap-on dildo (17.5%). Penile sleeve, penile injection and penile support device were rarely used (<10%).

**Clinical Implications:**

Data from our study can be used by clinicians to advise their clients, for example individuals with genital ablation interest who seek to maintain their sexual activities.

**Strengths & Limitations:**

Participants completed validated questionnaires. Data were collected online and could not be independently verified.

**Conclusions:**

A strong interest in genital ablation is often associated with a desire to be less sexual. Consistent with that is a high incidence of ED in this population. However, many men with exceptional interests in genital ablation nevertheless remain sexually active and use various strategies to maintain penetrative sex.

## Introduction

Erectile dysfunction (ED) is a common problem in aging men. ED is defined by the World Health Organization as an inability or marked reduction in the ability in men to attain or sustain a penile erection of sufficient duration or rigidity to allow for sexual activity.^1^ It is estimated that over 150 million men globally suffer from ED. In the U.S., about 52% of men age 40-70 report it.^2^ In a nationally represented U.S. sample of 1800 men (average age 47.5 years old) 24.2% reported ED.^3^ Incidence of ED increases with age. The aetiology of ED is multifactorial and may include psychological, physiologic, and iatrogenic causes.^2^^,^^4^

Sexual desire and interests vary considerably in those with interests in genital ablation. In a study of individuals who sought voluntary orchiectomies as many as 26% reported sexual dysfunction as a disadvantage from having genital ablation.^5^ A nearly equal amount (25%) saw becoming non-sexual (ie, decreased masturbation and/or thinking about sex) as an advantage to not having genitals, and 27.5% cited low libido and “no erections” as possible benefits of having non-functional testicles. Perhaps not surprisingly, more individuals who saw being non-sexual as an advantage of having non-funtional testicles chose not to be on androgen replacement therapy.^5^

In another study, sexual function scores varied between those who received voluntary orchiectomy, those who aspired for voluntary orchiectomy, and those who only fantasized about it, with orchiectomized individuals exhibiting the lowest sexual functioning and the fantasy group the highest.^6^ Gonadal hormones appear to play an important role in sexual function.^2^ Individuals who receive voluntary orchiectomy and take supplemental androgens experience a stronger sex drive, become aroused more easily, are more readily able to get and maintain erections, reach orgasm, and have better overall sexual functioning.^7^

Individuals with interests in genital ablations display a range of sexual attractions. In one study of eunuchs, nearly 18.4% reported being asexual, while 13.5% indicated they were bisexual.^7^ There was also a high percentage (15.3%) who were exclusively same-sex attracted.^7^ Aspiring eunuchs have similar attractions to eunuchs. Within one sample of 294 aspiring eunuchs, 9.9% reported asexuality, 13.9% bisexuality (Kinsey 3) and 22.1% homosexuality. Of note, individuals who only fantasize about genital ablation are less likely to endorse asexuality (3.2%) than aspiring eunuchs.^8^

Changes in sexual activity and attractions have been studied in the modern day voluntary eunuch population. In one study that included 198 physical eunuchs and 91 chemical eunuchs,^9^ a significant number remained sexually active and a large percentage experienced changes in their sexual attractions. For example, 37% reported having sex several times per week. In this sample, between 20-30% reported a change in their preferred gender(s) of attraction, sexual relationships, and fantasies. Approximately 8-11% became asexual. Individuals with Kinsey scores of 0 (exclusively opposite-sex attracted) and 6 (exclusively same-sex attracted) were the least likely to report these changes.^9^

In this study, we aim to answer the following questions: 1) How common is ED in assigned males with extreme interests in genital ablation? 2) What are their frequencies of sexual activities and what types of management strategies do they use? 3) What factors are associated with using these strategies? Data from this study can potentially be used by healthcare providers to advise clients on what strategies to use to retain sexual activities, in the presence of sexual dysfunction.

## Materials and methods

### Participants and procedures

The study protocol and questionnaires were approved by both the Institutional Review Board of California State University—Chico (IRB-2022-46), and the Eunuch Archive (EA) Steering Committee. Participants were recruited via the EA website (www.eunuch.org) between March 2022 and June 2023. This website is no longer active as of December 2024. The survey was built on the Research Electronic Data Capture (REDCap) and the survey URL was posted on the EA website.

We based our sample estimation on our third aim, ie, to determine what factors are associated with using sexual management strategies. To achieve that aim, we conducted multivariate logistic regressions with six independent variables. According to Green,^10^ a minimum of 97 participants is needed to conduct such analysis, with a power of 0.80 and a medium effect size.

Potential participants viewed the study information and could decide to participate in the study by clicking the study URL. Only those who consented to the study could access the full survey, which took approximately 30-45 minutes to complete. Participants received no compensation for their participation.

In total, 850 people consented to the study. We then excluded the following participants because: 1) 110 did not indicate their biological sex, 2) 7 were not biologically male (4 female, 3 intersex), 3) 61 did not answer the question about their castration status, 4) 258 did not provide their year of birth, 5) 27 provided ages that did not match with their birth year, 6) 13 were only interested in castration topic for academic reason, 7) 11 had unclear castration status. The final sample was 363 individuals.

### Measures

#### Demographics

Participants answered questions about their age, relationship status, education, income, gender, geographic location, and sexual attraction based on the Kinsey Scale (1948).^11^ For sexual orientation, participants could choose one of the following options: “X-Asexual or nonsexual (no interest in sexual activity with others),” “0-Exclusively heterosexual with no homosexual attraction,” “1-Predominantly heterosexual, only incidentally homosexual,” “2-Predominantly heterosexual, but more than incidentally homosexual,” “3-Bisexual (equal heterosexual and homosexual attraction),” “4-Predominantly homosexual, but more than incidentally heterosexual,” “5-Predominantly homosexual, only incidentally heterosexual,” and “6-Exclusively homosexual.” They were also asked about their use of hormone replacement therapy, medications, and recreational drug use, as well as whether they had chronic medical conditions.

Participants were asked about their castration status, with the following options: “Surgically castrated: both testicles have been removed and are no longer present”, “Physically castrated: both testicles have been destroyed, but remnants are still present. Use of burdizzo, injections of alcohol, etc. to destroy them”, “Nullified: Both penis and testicles have been removed”, “Chemically castrated: Currently using ‘reversible’ chemical castrating drugs such as Lupron, Androcur, high dose estrogen, etc.”, “Penectomized only: Penis has been removed, but testicles are still present”, “Formerly chemically castrated: Previously used chemically castrating drugs, but no longer do”, “Wannabe castrated: Would like to be castrated only and/or searching for way to be castrated only”, “Wannabe penectomized: Would like to be penectomized only and/or searching for way to be penectomized only”, “Wannabe nullified: Would like to have both testicles and penis removed, and/or searching for way to have both testicles and penis removed”, “Fantasy: Get pleasure (sexual or non-sexual) from thinking about castration or from ‘play castration’”, “Academic interest: Have no interest in being castrated yourself, but interested in the topic”, and “Other”. In addition, they were asked “have you had a testicle removed?” with the answer options “on both sides”, “one side”, and “no”. Participants were also asked “have you had your penis removed?” and they could select one of the following options: “full removal”, “partial removal”, and “no”.

Those, who were castrated (ie, chose the first four options above), were also asked to describe why they were castrated.

#### Male genital self image

Participants were asked to complete the Male Genital Self-Image scale.^12^ The scale consists of seven items, related to how men feel about their genitals. Each item can be rated from 1 (strongly disagree) to 4 (strongly agree). The scores were summed up to have a total score, with a higher score indicating more positive genital self-image.

#### Frequencies of sexual activities

To assess the frequencies of sexual activities, participants were asked: “On a typical week, how often do you watch porn?”, “On a typical week, how often do you masturbate?”, and “On a typical week, how often do you have sex with a partner(s)?” The answer choices for each question were: “more than once a day,” “once a day,” “several times a week,” “once a week,” and “rarely or never.”

#### Preferred sex roles

The survey included a question about preferred sex role: “During sex, what is your current preferred role?” The answer options were: “top (penetrative role; the one penetrating your partner’s vagina or anus),” “bottom (receptive role; being penetrated in the anus by your partner (e.g., using a penis, finger, sex toy, etc.),” “versatile (open to be on either role),” and “non-penetrative sex (e.g., oral, mutual masturbation).”

#### Erectile dysfunction and sexual management strategies

We included a question: “Do you have any erectile problems?” The responses were “0-25%,” “25-50%,” “50-75%,” “75-100% of the time,” and “does not have a full penis.” Additionally, we asked: “Have you used any of the following sexual aids for managing erectile dysfunction?” with seven options: “oral medication (e.g., Viagra, Cialis),” “vacuum erection device” (VED), “penile injection,” “penile implant,” “strap-on dildo,” “penile sleeve,” and “penile support device (e.g., the Elator).” The answer options were “yes” and “no” for each.

### Data analyses

#### Data analyses

Data were analyzed using SPSS (IBM, version 29). Descriptive statistics were used to summarize the demographics, frequency of sexual activities, preferred sex roles, ED frequencies, and their use of sexual management strategies. Male genital self-image was compared between participants who had and had not undergone castration by using the t-test. We conducted logistic regression analyses to determine how various factors were associated with the different sexual management strategies. In each model, the predictors were age, testicle removal status, penile removal status, preference to be insertive role, frequency of partnered sex, and frequency of erectile problems. Odds ratios (OR) for various predictors were reported. P < .05 was considered significant. Thematic analyses as guided by Braun & Clarke^13^ were conducted from responses of 94 participants who described their reasons for being castrated.

## Results

### Demographics

Table 1 shows the demographic data of our participants. The average age of our participants was 50.7 ± 15.6. The majority were White (90.9%), had university education (65.0%), of medium-to-high income (70.2%), and in either North America or Europe (91.7%). In term of their gender, 67.4% identified as men, and 18.5% identified as eunuchs. In addition, 12.7% were asexual, 28.1% had strong attraction to females, 30.0% had attraction to both sexes, and 29.2% had strong attraction to males. Some participants were on routine medications (38.1%), had chronic medical conditions (33.7%), and regularly used recreational drugs (11.1%).

**Table 1:** Demographic characteristics of participants.

Variables	N	%	Mean	SD
Age	363		50.7	15.6
Relationship status				
Single	145	40.3		
In a committed relationship	191	53.1		
In a casual relationship	15	4.2		
In multiple relationships	9	2.5		
Partners’ age	212		51.0	14.7
Sex of partner				
Male	70	32.6		
Female	148	68.8		
Intersex	2	.9		
Ethnicity				
White	330	90.9		
East Asian	14	3.9		
First Nations	6	1.7		
Hispanic	5	1.4		
South-East Asian	4	1.1		
South Asian	4	1.1		
Black	2	.6		
Middle-Eastern	2	.6		
Other ethnicities	6	1.7		
Gender				
Man	244	67.4		
Woman	14	3.9		
Eunuch	67	18.5		
Other genders	37	10.2		
Education				
Some high school or less	7	1.9		
Completed high school	58	16.1		
Vocational school	61	16.9		
Bachelor degree	118	32.8		
Master degree	80	22.2		
Doctoral degree	36	10.0		
Income				
Low	40	11.0		
Medium-low	68	18.8		
Medium	131	36.2		
Medium-high	93	25.7		
High	30	8.3		
Location				
North America	217	60.1		
Europe	114	31.6		
Asia	18	5.0		
Oceania	10	2.8		
Africa	2	.6		
Sexual attraction				
Asexual	46	12.7		
Kinsey 0	50	13.8		
Kinsey 1	52	14.3		
Kinsey 2	36	9.9		
Kinsey 3	53	14.6		
Kinsey 4	20	5.5		
Kinsey 5	35	9.6		
Kinsey 6	71	19.6		
On routine medication	134	38.1		
Have chronic medical condition(s)	120	33.7		
Regularly use recreational drug(s)	40	11.1		

### Genital modification and hormone use

As shown on Table 2, 28.7% of participants were castrated, 4.1% were formerly on chemical castration, 49% had a desire for castration and/or penectomy but had not gone through the procedure, and 18.2% only had genital ablation fantasies. Among the participants, 16.8% were on androgen therapy, 7.2% were on estrogens, 2.5% were on progesterone, and 1.1% were on other hormones. Some participants had their testicle(s) removed (23.6%) and 3.7% penectomized.

**Table 2:** Castration and genital modification status of participants.

Variables	N	%
Castration status		
Surgically castrated	61	16.8
Physically castrated	17	4.7
Nullified	10	2.8
Chemically castrated	16	4.4
Formerly castrated	15	4.1
Aspired to be castrated	113	31.1
Aspired to be penectomized	14	3.9
Aspired to be nullified	51	14.0
Fantasy	66	18.2
Hormone replacement use		
Androgens	61	16.8
Estrogens	26	7.2
Progesterone	9	2.5
Other hormones	4	1.1
Testicle removal		
None	275	76.4
One	9	2.5
Both	76	21.1
Penectomy		
None	344	96.4
Partial	1	.3
Full	12	3.4

Participants identified a number of reasons for castration. Gender or physical dysphoria was reported by 36 out of 94 (38.3%) participants. The second most commonly reported theme was medical reasons (eg, cancer treatment, chronic testicular pain, hypogonadism) that were reported by 24 (25.5%) participants. In addition, 19 (20.2%) reported their reason for electing castration was to lower their sexual desire. There were 16 (17%) who provided unspecified reasons (eg, simply wanting it, long term desire). Finally, 10 (10.6%) participants provided various reasons, including fetish, their partner wanting it, psychological reason, etc. We also noted that castrated participants reported a significantly higher genital self-image than those who were still intact (20.7 ± 4.9 vs. 19.4 ± 4.3; *t*(346) = 2.315, *d* = .277, P = .021).

### Sexual function, activities, and behaviours

**Figure 1:**
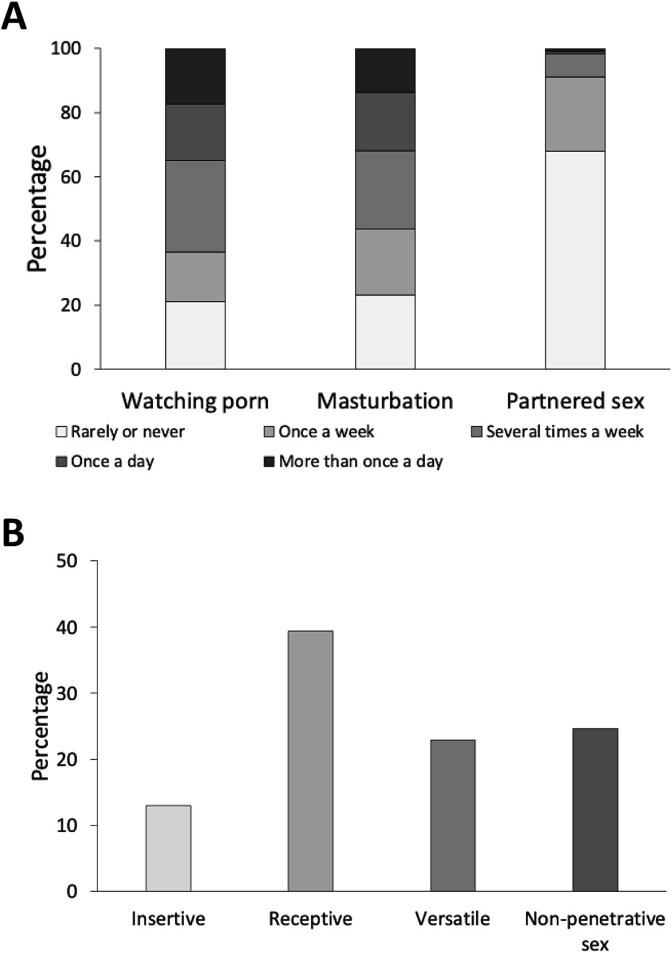
The frequencies of watching porn, masturbation and partnered sex (A), and sex role preferences (B) of participants in this study. Among them, 63.5%, 56.2% and 8.8% reported watching porn, masturbating and having partnered sex several times a week or more respectively. During partnered sex, 13% participants preferred to be the insertive role, whereas 39.4% and 22.9% preferred to be the receptive role and versatile respectively. In addition, 24.6% preferred non-penetrative sex.

As shown on Figure 1A, 63.5% of participants watched porn, 56.2% masturbated, and 8.8% had partnered sex several times a week or more often. During partnered sex (Figure 1B), 13% preferred to be the insertive, 39.4% preferred to be the receptive, 22.9% were versatile, and 24.6% preferred non-penetrative sex.

**Figure 2:**
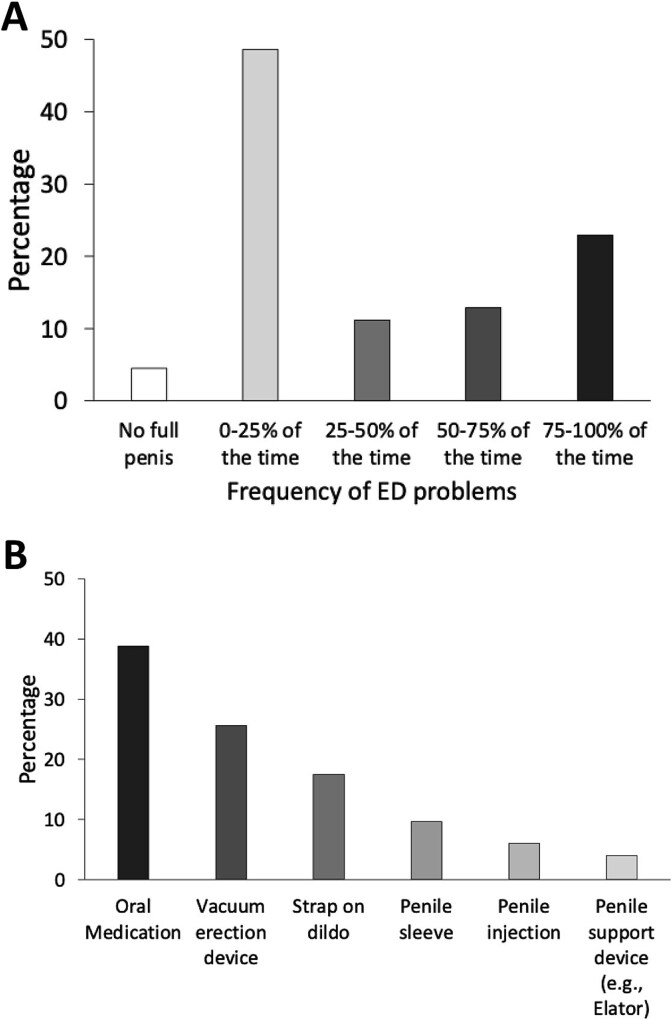
The frequencies of erectile dysfunction (ED) (A) and use of various sexual management strategies (B) of participants in this study. Among the respondents, 4.5% did not have a full penis. For the remaining, 11.2%, 12.8% and 22.9% reported having ED for 25-50%, 50-75%, and 75%-100% of the time. In term of sexual management strategies, 38.38%, 25.6% and 17.5% had used oral medication, vacuum erection device and strap-on dildo. Fewer than 10% had used penile sleeve (9.6%), penile injection (6%) and penile support device (4%).

In term of their erectile function (Figure 2A), close to half (48.6%) reported having minimal or no ED. For the remaining, 11.2% had ED 25-50% of the time, 12.8% had ED 50-75% of the time, and 22.9% had ED 75-100% of the time. To help them during sexual activities, the three most commonly used strategies were oral medication (38.8%), VED (25.6%), and strap-on dildo (17.5%). Penile sleeve, penile injection and penile support device were used by less than 10% of the participants. None of the participants reported having a penile implant.

### Predictors for using sexual management strategies

Table 3 shows how various factors were associated with using different sexual management strategies. Lower erectile function was associated with higher odds of using all ED management options or aids; ie, oral medication (OR = 1.52), VED (OR = 1.41), penile injection (OR = 2.14), strap-on dildo (OR = 1.50), penile sleeve (OR = 2.07), and penile support device (OR = 1.58). Older age was associated with higher odds of using oral medication (OR = 1.02) and VED (OR = 1.02), but lower odds of using a strap-on dildo (OR = 0.98). Having testicles removed was associated with higher odds of using oral medication (OR = 1.65), penile injection (OR = 2.45), but lower odds of using a penile sleeve (OR = .32). In contrast, penile removal was associated with higher likelihood of using strap-on dildo (OR = 2.38), and penile sleeve (OR = 8.94). Lastly, preference for the insertive role was related to lower odds of using a strap-on dildo (OR = 0.20), whereas frequency of partnered sex was related to a higher odds of using oral medication (OR = 2.52).

**Table 3:** Predicting variables for using various sexual management strategies.

Variables	OR	95% CI	
		Lower limit	Upper limit
*Oral medication*			
Age^*^	1.017	1.000	1.035
Testicle removal^**^	1.652	1.194	2.284
Penile removal	.656	.273	1.579
Preference for “top” role	1.177	.577	2.402
Frequency of partnered sex^***^	2.515	1.723	3.669
Erectile dysfunction^***^	1.526	1.245	1.871
*Vacuum erection device*			
Age^*^	1.019	1.000	1.038
Testicle removal	1.057	.753	1.482
Penile removal	1.453	.622	3.396
Preference for “top” role	1.235	.579	2.637
Frequency of partnered sex	.715	.476	1.074
Erectile dysfunction^**^	1.412	1.149	1.735
*Penile injection*			
Age	1.032	.991	1.074
Testicle removal^**^	2.448	1.428	4.197
Penile removal	1.334	.309	5.760
Preference for “top” role	2.869	.683	12.049
Frequency of partnered sex	1.451	.789	2.670
Erectile dysfunction^***^	2.144	1.362	3.377
*Strap-on dildo*			
Age^*^	.976	.956	.996
Testicle removal	.887	.589	1.336
Penile removal^*^	2.383	1.025	5.543
Preference for “top” role^*^	.199	.046	.868
Frequency of partnered sex	1.204	.828	1.751
Erectile dysfunction^**^	1.495	1.167	1.916
*Penile sleeve*			
Age	.992	.963	1.020
Testicle removal^*^	.317	.127	.793
Penile removal^**^	8.935	2.390	33.402
Preference for “top” role	.609	.166	2.241
Frequency of partnered sex	1.565	.980	2.499
Erectile dysfunction^***^	2.067	1.465	2.917
*Penile support device*			
Age	.989	.951	1.027
Testicle removal	1.372	.729	2.580
Penile removal	1.761	.478	6.493
Frequency of partnered sex	1.107	.555	2.207
Erectile dysfunction^*^	1.576	1.007	2.468

^*^Significant predictor, P < .05, ^**^P < .01, ^***^P < .001. OR = Odds ratio

## Discussion

There are several main findings from this study. First, watching porn and masturbation are common, but partnered sexual activities are not frequent in assigned male individuals with genital ablation interests, which may be due to the presence of ED for many of them. Second, during partnered sex, the majority preferred either a receptive or versatile role, and they may use an aid for penetrative sex, such as an oral medication (presumably a phosphodiesterase inhibitor), the VED, and/or a strap-on dildo. Third, the most common factor for using a sexual aid was ED frequency, but age, genital ablation, preferred sex role, and sexual frequencies may also affect their openness in using various strategies. Data from our study can be used by clinicians to advise their clients, for example individuals with either erectile dysfunction or genital ablation interests who seek to maintain their sexual activities.

### Demographics

This sample consisted predominantly of individuals who were castrated or aspired to be castrated. A high proportion identified as asexual (12.7%, n = 46), bisexual (14.6%, n = 53), and same-sex attracted (19.6%, n = 71) on the Kinsey scale, similar to other samples of eunuchs and aspiring eunuchs.^7^^,^^8^ In keeping with these past studies, many individuals with interests in genital ablation identify as men, while a substantial proportion identify as eunuchs (18.5%, n = 67) or genders other than that assigned at birth (14.1%, n = 51), illustrating the intersection between genital ablation interests and gender identity variance.

### Genital modification and hormone use

Castrated participants in this sample reported a variety of reasons for seeking genital ablation. Gender or physical dysphoria (n = 36, 38.3%) was the most commonly cited reason for seeking castration. This is in keeping with a recent study finding that nearly half (n = 87, 46.3%) of those who desired castration did so because it aligned with their gender or physical embodiment goals.^5^ It is not surprising then, that castrated participants in our sample reported much higher genital self-image than those who had not undergone genital ablation.

For those on hormone therapy, a majority of castrated participants chose either androgens (n = 61, 16.8%) or estrogen (n = 26, 7.2%). This finding is also not surprising given that a majority of the total sample either identified as men (n = 266, 67.4%) or eunuchs (n = 67, 18.5%). Prior studies have found that eunuchs often maintain a masculine gender expression and, should they desire HRT, choose androgens as their preferred hormone replacement.^5^

### Prevalence of erectile dysfunction

In this study, around 50% of participants (average age 50.7 years old) reported having some levels of ED. This finding is higher than the general population of similar ages. Another study in the United States.^3^ showed that ED was reported in 24.2% of over 1800 men (average age 47.5 years old). However, our finding is not surprising, given that many had received treatments that can impair their erectile functions; notably, 28.7% were castrated.

### Sexual function, activities, and behaviours

Masturbation and engagement with pornography, but not partnered sex, were relatively common in this sample. This may be due in part to many castrated individuals in this study being on HRT (androgens or estrogens). The significant number of participants who rarely or never engaged in partnered sex (about 70%) may be related to high levels of ED, which are not necessarily associated with sexual desire. Past research of individuals who sought voluntary orchiectomies, showed that as many as 26% reported sexual dysfunction as a disadvantage of genital ablation.^5^ As such, many eunuchs and aspiring eunuchs may desire to be non-sexual with fewer erections as a goal. However, our data illustrate the importance of clinicians not assuming that all individuals who seek or desire genital ablation necessarily have the same goals for sexual functioning.

### Use of sexual management strategies

Given the high prevalence of ED in our sample, the use of sexual management strategies is common, especially oral medication and the VED. These two strategies are frequently prescribed in men with ED, for example after receiving prostate cancer treatment.^14^ However, unlike in the prostate cancer patient population (eg, 2.9% in the study by Duthie et al. (2021)), 17.5% of individuals in this sample had used a strap-on dildo. This finding suggests that assigned male individuals with genital ablation interests are more open to using diverse sexual management strategies. While using a strap-on dildo is not common among the general population, peoples’ willingness to try it may change when they receive appropriate information about it.^15^

None of our participants had used a penile implant. This is expected because the majority have interests in genital ablation and conversely little or no interest in a surgically implanted prosthesis. Indeed, many had reported that they sought treatment to have lower sexual desire and fewer erections.^5^ Furthermore, only 13% of our participants had a strong preference to be the insertive (penetrating) partner during sex.

The use of penile sleeve and penile support device is rare. In our study, 9.6% and 4% reported having used a penile sleeve or a penile support device respectively. These proportions are higher than the data from prostate cancer patients who experience ED. For example, Duthie et al. (2021) showed that only 1.4% had used a penile sleeve or penile support device.^14^

Both our study and the study by Duthie et al. (2021) showed that strap-on dildos, penile sleeves, and other penile support devices are not commonly used by assigned males with ED.^15^ This may in part be because they are predominantly marketed as sex toys, and may be considered inferior strategies for restoring penetrative sex. There is also a lack of objective assessment in the medical literatures on the experience of men who had used these strategies, so clinicians may be reluctant to encourage their use for patients with ED. A recent study with a small sample size showed though that a penile support device, the Elator, generally received positive feedback from men with severe ED and their partners.^16^

### Limitations

There are several limitations in this study. Not all participants reported their reasons for castration). The data were collected from a website for individuals with extreme interests in genital ablation, so the finding cannot be generalizable to the general population. All data were collected online, so we cannot confirm all facts and there may be recall biases in the responses. In order to minimize the impact of inaccuracies, we removed data from participants whose age could not be matched with their birth year. Given the various available sexual management strategies, the options we asked our participants were limited. For example, we did not ask if they used vibrators or anal toys (eg, butt plugs, anal beads) for solo or partnered sexual activities. This information may be relevant to those who do not prefer the insertive role.

## Conclusion

Among assigned male individuals with genital ablation interests, partnered sexual activities are not frequent. This may be partially because about 50% had some levels of ED. When they engage in partnered sex, the majority preferred either a receptive or versatile role. They may also use a sexual aid for sexual activities, with oral medications, the VED, and a strap-on dildo being most commonly used strategies. The main factor for using these strategies was ED frequency, but other factors—age, genital ablation, sexual role, and sexual frequencies—may also influence the willingness of the men to use these strategies. Data from our study can be used by clinicians to advise their clients, for example individuals with erectile dysfunction or genital ablation interests who seek to maintain their sexual activities.
